# Albufera Lagoon Ecological State Study Through the Temporal Analysis Tools Developed with PerúSAT-1 Satellite

**DOI:** 10.3390/s25041103

**Published:** 2025-02-12

**Authors:** Bárbara Alvado, Luis Saldarriaga, Xavier Sòria-Perpinyà, Juan Miguel Soria, Jorge Vicent, Antonio Ruíz-Verdú, Clara García-Martínez, Eduardo Vicente, Jesus Delegido

**Affiliations:** 1 Image Processing Laboratory, University of Valencia, 46980 Valencia, Spain; barbara.alvado@uv.es (B.A.); luisalre@alumni.uv.es (L.S.); javier.soria-perpina@uv.es (X.S.-P.); jorge.vicent@uv.es (J.V.); antonio.ruiz@uv.es (A.R.-V.); clara.garcia-martinez@uv.es (C.G.-M.); 2 Space Agency of Peru (CONIDA), Lima 15046, Peru; 3 School of Transportation Science and Engineering, University of Beihang, Beijing 102200, China; 4 Cavanilles Institute of Biodiversity and Evolutionary Biology, University of Valencia, 46980 Valencia, Spain; juan.soria@uv.es (J.M.S.); eduardo.vicente@uv.es (E.V.)

**Keywords:** remote sensing, water quality, ecology, PerúSAT-1, hypertrophic

## Abstract

The Albufera of Valencia (Spain) is a representative case of pressure on water quality, which caused the hypertrophic state of the lake to completely change the ecosystem that once featured crystal clear waters. PerúSAT-1 is the first Peruvian remote sensing satellite developed for natural disaster monitoring. Its high spatial resolution makes it an ideal sensor for capturing highly detailed products, which are useful for a variety of applications. The ability to change its acquisition geometry allows for an increase in revisit time. The main objective of this study is to assess the potential of PerúSAT-1′s multispectral images to develop multi-parameter algorithms to evaluate the ecological state of the Albufera lagoon. During five field campaigns, samples were taken, and measurements of ecological indicators (chlorophyll-a, Secchi disk depth, total suspended matter, and its organic-inorganic fraction) were made. All possible combinations of two bands were obtained and subsequently correlated with the biophysical variables by fitting a linear regression between the field data and the band combinations. The equations for estimating all the water variables result in the following R^2^ values: 0.76 for chlorophyll-a (NRMSE: 16%), 0.75 for Secchi disk depth (NRMSE: 15%), 0.84 for total suspended matter (NRMSE: 11%), 0.76 for the inorganic fraction (NRMSE: 15%), and 0.87 for the organic fraction (NRMSE: 9%). Finally, the equations were applied to the Albufera lagoon images to obtain thematic maps for all variables.

## 1. Introduction

Coastal lagoons are often formed when a sandbar encloses a former bay [1]. These wetland ecosystems, like other lentic water bodies, are highly sensitive to anthropogenic pressures and impacts. These can alter their ecological functionality and compromise their uses for human activities [2]. The ecological status of water bodies is the central concept of the European Water Framework Directive (WFD), relating the actual status of a given water body with a set of reference conditions that represent its ecological optimum [3]. The status is assessed based on a set of biophysical variables obtained from conventional in situ monitoring. Despite not being included in the initial formulation of the WFD, remote sensing techniques are increasingly being used to assess, in a systematic and synoptic manner, some of those biophysical variables. Optical satellite sensors can retrieve some key indicators of a water body’s ecological status, including chlorophyll-a (Chl-*a*) concentration, the total suspended matter (TSM), the absorption coefficient of dissolved organic colored matter, and the water transparency, measured as the Secchi disk depth (SDD) [4].

The Chl-*a* concentration is commonly used as an indicator of the phytoplankton biomass since almost all species contain the key photosynthetic pigment. Chl-*a* concentration is also an indicator of the eutrophication process, caused by increased nutrients (N, P and other elements), which is often associated with human activities. Therefore, Chl-*a* acts as a connector between nutrient concentration and primary production [5]. This biophysical parameter has two distinct absorption peaks, centered in vivo at wavelengths of 440 nm and 675 nm [6].

Particles present in the water column absorb and scatter light. A higher concentration of particles leads to increased scattering and therefore an increase in the turbidity of water bodies [7]. The TSM determined by filtration of water samples consists of inorganic particles (minerals, sand, etc.) as well as other organic particles (phytoplankton, detritus, etc. [8]. An increase in the TSM increases scattering and therefore the reflectance of a water body. This effect is especially noticeable in spectral regions where the absorption of other optically active constituents of the water body is low. There is a direct positive correlation between the reflectance in the red spectral region and the concentration of suspended solids in water due to their particle dispersion properties [9]. The Secchi disk depth (SDD) is used in limnological studies to estimate the extinction of light in water [10]. The SDD is inversely related to the amount of suspended matter in the water [11]. As water clarity decreases, brightness in the red spectral region usually increases [9].

The majority of water quality studies are conducted through in situ measurements and subsequent chemical analysis in laboratories. These methods allow researchers to gain insights into aquatic ecosystems, forming a basis for long-term monitoring to assess the ecological situation and identify trends in water bodies. The water reflectance is measured to analyze its optical properties and derive concentrations of optically active components present in the water column. The use of remote sensing as a tool for monitoring the ecological quality of water masses has great potential as a complementary strategy in an indirect, remote, frequent, and continuous manner over time. Remote sensing methods are often based on semi-empirical or empirical algorithms derived from the statistical regression, between the reflectance values of the water in different spectral ranges and the concentration of the water constituents [12]. Many authors have successfully used remote sensing technologies to map different water quality variables: TSM in the Gironde estuary [13]; TSM in the Frisian Lakes [14]; Chl-*a*, SDD and TSM in the Albufera of Valencia and natural ponds [15]; and Chl-*a* and SDD in the Albufera of Valencia and different Spanish reservoirs [16]. The small size and complex shapes of most inland waters require the use of sensors with high spatial resolution. Among them, Landsat’s sensor is the go-to for these studies because it has a relatively high spatial resolution (30 m) and a long period of archived data. Some of these studies have even been done in the Albufera of València [17,18]. On the other hand, Landsat’s sensor has a limited spectral resolution and low temporal resolution, which makes it difficult to accurately determine some of the water quality variables. ESA’s Sentinel-2 (S2) mission has outstanding characteristics for measuring inland waters’ biophysical variables. The spatial resolutions of 10, 20, and 60 m, coupled with a revisit frequency of 5 days, significantly enhance the capabilities of the Landsat mission. Recent studies have successfully used S2 images to measure phycocyanin [19] and turbidity and TSM [20]. SPOT satellites have spatial resolutions of less than 10 m. Despite having considerable bandwidth, studies have developed satisfactory results measuring Chl-*a* and TSM in lakes [21] or Chl-*a*, SDD, and total phosphorus in reservoirs [22].

In 2016, the Peruvian Space Agency launched an operational Earth observation mission, named PerúSAT-1, with the main objective of monitoring and evaluating natural catastrophes. This satellite features a high spatial resolution NAOMI sensor [23] and operational agility, which together enable enhanced spatial and temporal resolution for diverse surface monitoring. Building on these capabilities, this study aims to determine whether PerúSAT-1 images can be used to generate water inland quality assessment products that accurately describe the ecological state of the Albufera lagoon (Valencia, Spain). For this, we propose the following specific objectives: (1) atmospherically correct the images to synthesize advanced products, (2) develop algorithms that can estimate the optical properties of water through the combined use of satellite images and a database of different sampling in the Albufera lagoon, and (3) analyze the suitability of the PerúSAT-1 satellite to monitor the quality of the lagoon remotely and continuously over time.

## 2. Materials and Methods

### 2.1. Study Area

**Figure 1:**
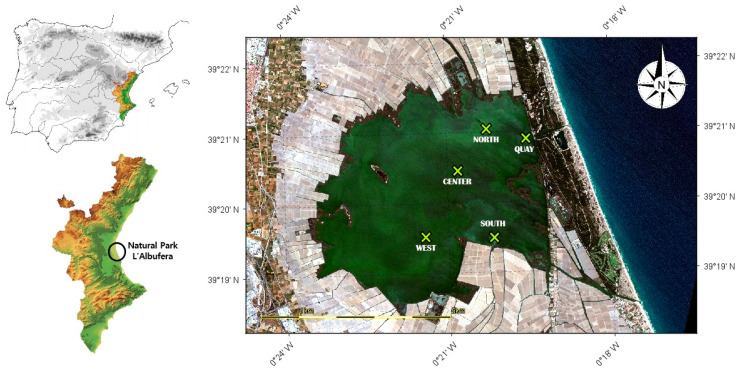
Study area location *L’Albufera de València*. Green dots are the sampling points.

The Natural Park of *L’Albufera de València* is a coastal wetland located on the south of Valencia city—east of the Iberian Peninsula (Figure 1). It covers an area of approximately 21,000 hectares (ha), comprising 14,000 ha of marshes and 2800 ha of shallow lake.

Being in a coastal alluvial plain, the lagoon is formed by the contributions of the Turia and Júcar rivers to the Mediterranean Sea [24]. With an approximate depth of 1.3 m, the lagoon has unquestionable importance for its significance in regulating water flow in the rice fields. The marshes are mainly dedicated to rice cultivation and occupy the largest part of the park. This area has expanded continuously due to artificial clogging to increase the cultivation area. The local water council is responsible for regulating the hydrology of the area, with inflow occurring via irrigation channels [19]. As summarized in [25] and detailed in [26], the outflow to the Mediterranean Sea is through sluice gates located at the outlet canals, following an annual cycle. From November to the beginning of January, the sluice gates are closed, leaving the rice fields flooded. At this time, the water level of the lagoon is about 20–30 cm above normal. The flooded rice fields are emptied between January and February, with the water flowing out to the sea through the opened sluice gates. Furthermore, the water level in the lagoon also declines. The rice fields are kept dry until the beginning of May, when they are flooded again for rice cultivation. During rice cultivation, the sluice gates are partially closed to maintain a mean water flow and a level no more than twenty centimeters, suitable for rice.

Due to its high ecological value, it has been declared a Natural Park, a Special Protection Area for Birds (SPA), and was included in the List of Wet Areas of International Importance of the RAMSAR Conference. It is the second most important wetland in the Iberian Mediterranean, after the Ebro Delta [27,28]. The lagoon is currently in a hyper-eutrophic state due to the dumping of large volumes of urban and industrial wastewater through ditches since the 1970s [29]. Furthermore, agricultural returns are one of the primary sources of nutrients in the lagoon [30].

### 2.2. Field Campaigns and Laboratory Analysis

The most effective method for calibrating and validating remote sensing algorithms is to collect field data simultaneously with the passage of a satellite over the study area [31]. Validation is an essential process to ensure that satellite observations are adequate for the intended use of the data [32]; therefore, it is important to define an acceptable time window for in situ measurements [33]. Ref. [34] states that the time interval between satellite passage and sample collection can be extended to three days. To test the stability of remote sensing reflectances (Rrs), ref. [35] proposed a statistical analysis using a Student’s *t*-test to compare water spectra at both dates, using Sentinel-3 images. Furthermore, meteorological changes were verified [36] as values of temperature, wind, and rain between image and sample data were consulted on the open data web page of the Spanish State Meteorological Agency (https://opendata.aemet.es/centrodedescargas/productosAEMET) (accessed on 6 June 2024).

From 2018 to 2023, five field campaigns were carried out over the Albufera lagoon, obtaining 22 georeferenced water samples, at points selected from the study of [37]. The “north” and south” points are located near the outlet of irrigation ditches that bring water from the fields to the lagoon. On the other hand, “center” and “west” points are located in more central areas of the lagoon. Finally, the “quay” point is located next to the sluice gates that connect the lagoon with the Mediterranean Sea. Usually, sampling in the lagoon is done at the same five points, but the “quay point” is not always sampled. The field campaigns were planned in accordance with the methodology set out by [38]. Cloud-free PerúSAT-1 images were selected from the closest available date (Table 1).

On each campaign, the physicochemical water variables were measured over different locations of the lagoon (4 to 5 points). The SDD, water temperature, and conductivity were the in situ measured variables. The other variables were measured in the laboratory from the water samples. Secchi disk depth was used for measuring water transparency. A Secchi disk is a standard 20 cm diameter disk that is plunged vertically to the depth at which it is no longer visible. SDD is therefore measured as the mean depth at which the disk disappears and reappears [39].

The samples were collected from the superficial water layer in the sampling points and immediately refrigerated at 4 °C in dark conditions for later transport to the laboratory. Chl-*a* samples were filtered through Whatman GF/F glass fiber filters, with a pore size of 0.4–0.6 µm, and extracted according to [40]. The results were then calculated using spectrophotometry in line with the methodology set out by [41]. In addition, gravimetry [42] was performed to determine the TSM. This method begins with drying the filter (Whatman 934-AH filter—1.5 µm pore) containing the particulate material at 105 °C, thus obtaining the value of TSM. Subsequently, the same filter is heated for 6 h at 460 °C to obtain the value of the particulate inorganic matter (PIM). The final step is to calculate the particulate organic matter (POM) by subtracting the previously obtained values. All the variables were correlated using a normality test combined with Pearson correlation (parametric correlation) in case of normality or Spearman correlation (non-parametric correlation) in case of non-normality.

### 2.3. PerúSAT-1 Images

PerúSAT-1 is the first remote sensing satellite operated by the Peruvian Space Agency. The system includes a last-generation optical satellite that observes the Earth through the NAOMI (New AstroSat Optical Modular Instrument) optical camera, which has a very high resolution. Table 2 shows the spectral range and spatial resolution of each band.

Multispectral bands with a 2.8 m spatial resolution allow more detailed mapping of biophysical parameters in water than larger pixel sizes. Furthermore, they allow measurements to be taken in narrow areas (i.e., canals connecting rice fields with the lagoon; quay located in the sluice gates) and improve the analysis in areas where there is an inflow of clean water. On the other hand, PerúSAT-1 is not a fixed-pointing satellite, like Sentinel-2 or Landsat. This enables changing observation geometries for acquiring multi-angle images over the same target and increasing the revisit period to obtain images within 3 days, over Perú or countries near the equatorial line, a very important feature when it is required to study a certain event in a specific place. Similar to a heliosynchronous satellite, the vertical passage times over any point on Earth are constant.

PerúSAT-1 imagery is delivered at various levels of geometric and spectral processing. Level 3 (L3) corresponds to a georeferenced and orthorectified four-band multispectral image. All PerúSAT-1 images are provided as digital numbers (DNs), which represent raw data measured directly by the satellite at the top of atmosphere (TOA), thus requiring conversion to reflectance values for analysis.

#### 2.3.1. Atmospheric Correction

Due to the low reflectance of water, atmospheric correction is a key data processing step for water quality studies through remote sensing [43]. Atmospheric correction is the process that removes the atmospheric influence on the total radiation entering the sensor, i.e., the compensation of absorption and scattering effects caused by gas molecules and aerosols [44]. Atmospheric correction is mainly divided into two sequential steps: (1) the characterization of the atmospheric conditions (namely gas concentration and aerosol conditions) at the time of satellite overpass and (2) the retrieval of surface reflectance. For the first step, we rely on the total water column, total ozone column, and aerosol optical depth at 550 nm extracted from the Copernicus Atmosphere Monitoring Service re-analysis products. These atmospheric parameters underwent spatial and temporal interpolation to match the satellite’s passage over the lagoon. Re-analysis products were used instead of a satellite-based retrieval algorithm given the inadequate spectral band configuration in PerúSAT-1 (i.e., four coarse bands placed in atmospheric windows). For the second step, we relied on the inversion of an atmospheric radiative transfer model (6SV2.1 in our case [45]). The 6SV was run with the atmospheric and geometric conditions corresponding to the PerúSAT-1 observations to derive the atmospheric reflectance and transmittances. The surface reflectance, ρ, was derived under the assumption of Lambertian surface and by solving for ρ in Equation (2), which requires a pre-computation of $L_{TOA}$ using Equation (1):

(1) $$L_{TOA}=\frac{DN_{\left(x,y,b\right)}}{GAIN_{b}}+BIAS_{b}$$

where *x*, *y*, and *b* are the row, column, and band indices, respectively, of PER1 pixels; $L_{TOA}$ represents the TOA radiance; DN is the pixel’s digital number, ${GAIN}_{b}\;is$ the radiometric gain coefficient for band b; and $BIAS_{b}$ is the offset radiometric calibration coefficient for band *b*.

(2) $$L_{TOA}=L_{0}+\frac{I_{0}T_{g}T^{↓↑}\rho }{\pi \left(1-S\rho \right)}$$

where $L_{0}$ is the atmospheric path radiance, $I_{0}$ is the solar irradiance, $T_{g}$ is the gas transmittance (mainly O_2_, O_3_, and H_2_O), $T^{↓↑}$ is the total (direct and diffuse) downward and upward transmittance, and S the spherical albedo.

#### 2.3.2. Spatial Resolution Capacity

The spatial resolution of satellite images is one of the essential aspects of remote sensing, as the level of detail in an image is directly dependent on this factor. According to [46], spatial resolution determines the smallest object or feature in the real world that can be captured and thus the suitable applications for this product. A comparison has been made with an S2 image to study the spatial resolution capacity of the PerúSAT, using the images acquired on 20 April 2023. The S2 image processing was done according to [47], using the ESA *Sentinel Application Platform* (SNAP). The formulas used to retrieve each parameter in the S2 images have been developed in different studies specifically with data from the Albufera of Valencia and are summarized in Table 3.

### 2.4. Retrieval Algorithms

A multispectral image contains reflectance data in different interval bands. These bands can be combined through an arithmetic operation to obtain the correlation between the field data and the satellite sensor data [50]. The images were processed using the SNAP program. In this processing stage, regions of interest (ROIs) of 100–150 pixels are established around each of the sampling points to deduct the average reflectance of each band. These spectra and the field data are used to create a database for calibrating and validating spectral indices.

The Spectral Index (SI) tool in the ARTMO (Automated Radiative Transfer Models Operator) package [51] allows all possible band combinations to be defined and evaluated. Mathematical combinations of measured reflectance at different wavelengths are correlated with in situ parameters, using the previously created database. To obtain different possible combinations of bands, we have used the simple ratio (SR: R1/R2) and normalized difference (ND: (R2 − R1)/(R2 + R1)) formulas, correlating the data through different fitting functions (linear, exponential, power, logarithmic, and polynomial). All the data of the biophysical variables obtained in the different field campaigns were used, and with the cross-validation tool, all the data were used to create different sets for training and validation. The K-fold method divides the data into 10 parts, which are used for both calibration and validation of the model. Each time, the model uses nine parts for calibration and one to validation, creating different training sets, and the result is an average of all the statistical results. All the best possible combinations were finally calibrated using a linear regression between the field data and the band combinations, searching for the best fitting function. Validation was carried out using statistical variables based on observed and estimated data: coefficient of determination (R²), root-mean-square error (RMSE), and normalized RMSE (NRMSE), mean absolute error (MAE), and bias. Using the most accurate algorithms, thematic maps for all the variables were created using the SNAP program, which allows us to visualize the spatial distribution of all the variables. To adapt to the study area, a water mask was created considering the low reflectivity present in the infrared band (B4), so that the algorithms could be applied and the results extracted only from the lagoon. Finally, by applying different color palettes, we can observe the spatial and temporal changes through the seasons.

## 3. Results and Analysis

### 3.1. Atmospheric Correction

Once the atmospheric correction is applied, scattering and absorption effects are removed, and we then have a clear image representing the surface reflectance.

**Figure 2:**
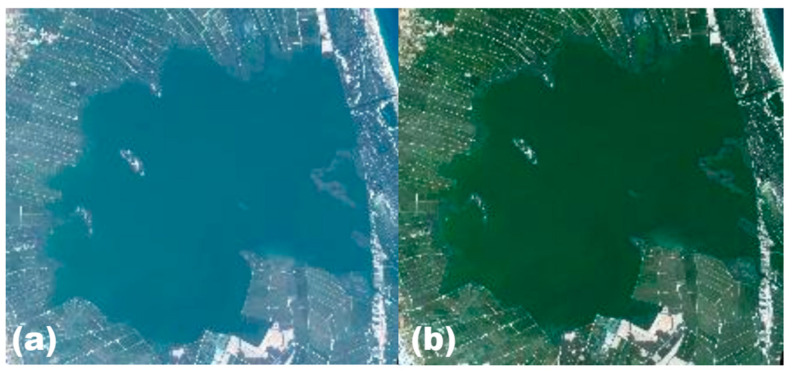
PerúSAT-1 images over the Albufera lagoon. Image TOA (**a**), without atmospheric correction, and image BOA (**b**), with atmospheric correction.

Figure 2 illustrates the atmospheric correction process by comparing an image with reflectance measured at TOA and bottom of atmosphere (BOA), i.e., after atmospheric correction. The image corresponds to the study area, dated 22 May 2018. This figure shows how the haze effects of aerosols and molecular scattering are reduced, especially at lower wavelengths, and how the surface reflectance data have a better contrast of surface characteristics.

**Figure 3:**
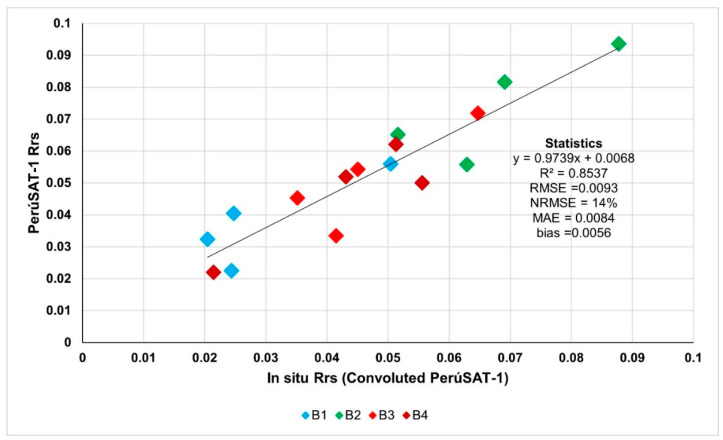
Validation of atmospheric correction data.

Atmospheric correction has been validated with the Rrs obtained in situ. For this, the Rrs obtained during the 20 April 2023 field sampling has been convoluted to PerúSAT-1 bands. Figure 3 shows the correlation between PerúSAT-1 product data and Rrs in situ convoluted, with a relatively low error (RMSE = 0.093).

### 3.2. Retrieval Algorithms

**Figure 4:**
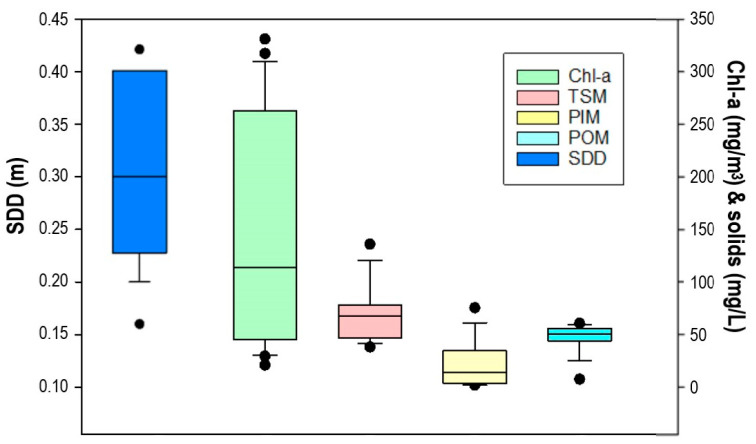
Boxplot of the values range for the water quality parameters. The box bounds the interquartile range (IQR: 25–75 percentile), the horizontal line inside the box indicates the median, and the error bars indicate the 90th above and 10th below percentiles. Dots indicate the outliers.

The database of each variable was displayed in a boxplot to understand the distribution of sample values over the variables ranges (Figure 4). The variables showed a skewed distribution, with the mean at the lowest values in Chl-*a* and PIM and at the highest values in TSM. The POM variable showed a lower distribution of the data.

In an initial analysis, the cross-correlation between all the variables was established in order to understand their relationships (Table 4). Chl-*a* and TSM show a strong correlation between them, indicating that TSM is, in most cases, mainly composed of phytoplankton. Both variables have a strong inverse correlation with SDD, since the transparency of the water in the Albufera lagoon is reduced as their concentrations increase. The strong correlation between Chl-*a* and TSM indicates that most of the suspended solids are due to phytoplankton, so that phytoplankton has a predominant role in the turbidity of the Albufera lagoon.

In reference to the development of algorithms, the results for each variable will be detailed as follows: a table showing the five best band combinations providing the lowest RMSEs, as well as the statistical errors associated with the measured and estimated values, and a plot of the best correlation between the band combinations and the variable values.

**Figure 5:**
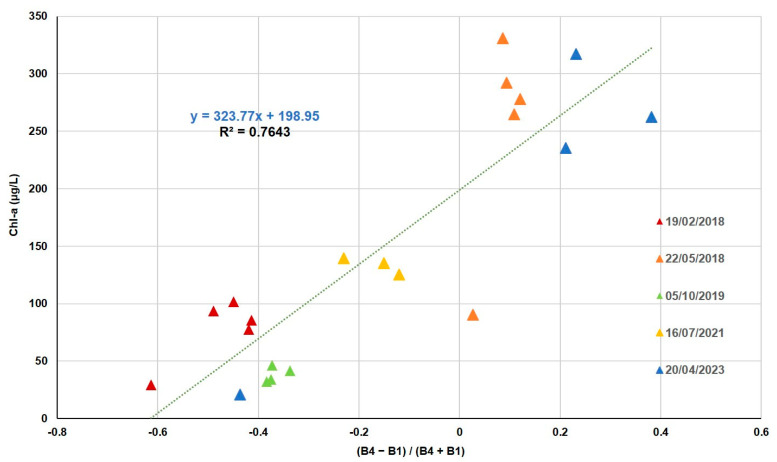
Chl-*a* in situ as a function of ND (B4 − B1)/(B4 + B1).

The 21 Chl-*a* values obtained from laboratory analysis range from 21 µg/L to 331 µg/L. Different indices combining two bands have normalized relative errors below 20% between the values measured in situ and the Rrs. ND using band 1 (blue) and band 4 (near infrared) is the combination that reaches the best correlation (Table 5) with the Chl-*a* fitted by the method of least squares to a linear fitting function (Figure 5).

The highest Chl-*a* values occurred in spring 2018 and 2023, as shown in Figure 5.

With respect to the 22 SDD values, they are about 0.16 m and 0.42 m. In this case, the validation of the Rrs values with the in situ values resulted in the lowest error at 9. The best correlation with SDD (Table 6) was obtained by ND using band 1 (blue) and band 4 (near infrared), the same combination obtained for Chl-*a*.

**Figure 6:**
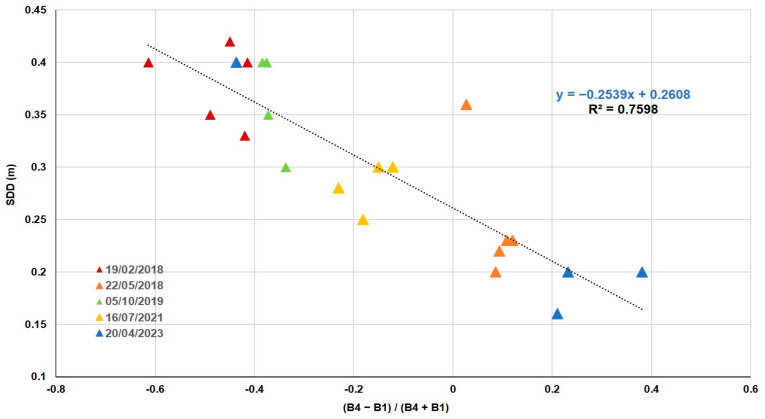
SDD in situ as a function of ND (B4 − B1)/(B4 + B1).

In Figure 6, we can observe the equation of the best linear fitting function with the method of least squares. It can be seen that the lowest values of SDD are those in which the values of Chl-*a* have reached their maximum (Figure 6), in the spring of 2018 and 2023.

**Figure 7:**
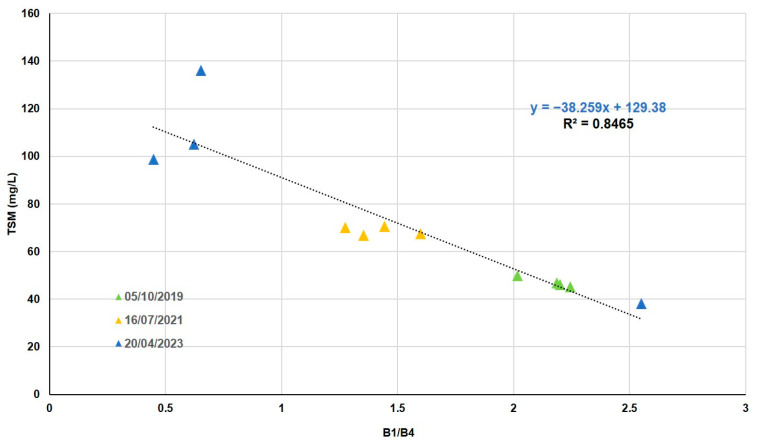
TSM in situ as a function of SR (B1/B4).

The 12 TSM values in the lagoon vary from 39 mg/L to 136 mg/L. Combining all the bands in different ways shows us that the best error is around 8%. The SR of bands 1 and 4 gave the best correlation with the TSM data measured in situ (Table 7). This correlation is obtained by the method of least squares to a linear fit function (Figure 7).

As can be seen in Figure 7, the increase in the amount of TSM present in the lagoon in the spring is noticeable in relation to the different time periods of the images.

The 12 inorganic fraction values vary between 2 and 75 mg/L. The combinations of all the bands give errors of about 15% between the in situ values and those measured by the satellite. ND with band 3 (red) and band 1 (blue) is the combination that reaches the best correlation with the PIM field data (Table 8).

**Figure 8:**
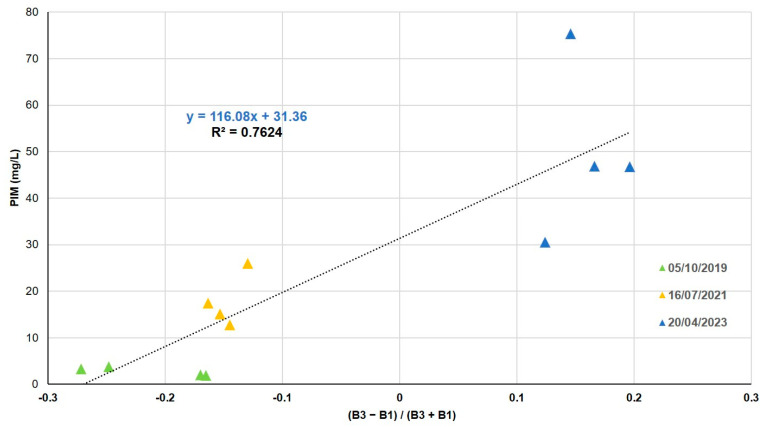
PIM in situ as a function of ND (B3/B1).

This correlation is obtained by the method of least squares to a linear fit function (Figure 8). As TSM, it shows the majority of PIM in spring.

We could find in 12 values an organic fraction of the TSM from 7 to 60 mg/L. Different combinations using all the bands give us errors near 8%. With the SR of bands 3 (red) and 4 (near infrared), we obtained the best combination that reached the best correlation with POM field data (Table 9).

**Figure 9:**
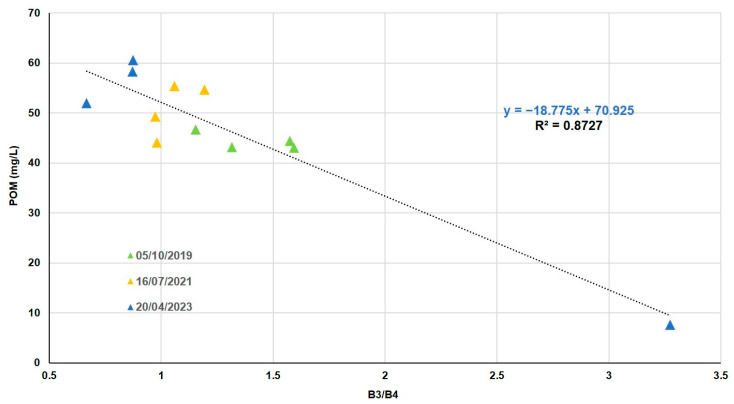
POM in situ as a function of SR (B3/B4).

The best fitting function is obtained by fitting with the method of least squares to a linear fitting function (Figure 9). It also reproduces an increase in POM concentrations in spring. In addition, when comparing Figure 8 with Figure 9, we can observe the predominance of the organic fraction in the TSM of the lagoon.

Table 10 is a summary of the best algorithms for each variable studied, using the entire field database. It is possible to see the range of data used, the amount of data used (*n*), the algorithms obtained, the R^2^, and the validation statistics (RMSE and MAE are in the corresponding units of each variable).

The model developed for the estimation of Chl-*a* is the one with the highest MAE, so it should be taken into account that the error is significant. The rest of the models have lower MAE values, so the predictions are closer to the observed values. As for bias, the models developed for Chl-*a* and POM underestimate the values, and those developed for SDD, TSM, and PIM overestimate them.

### 3.3. Thematic Maps of the New Algorithms for PerúSAT-1

**Figure 10:**
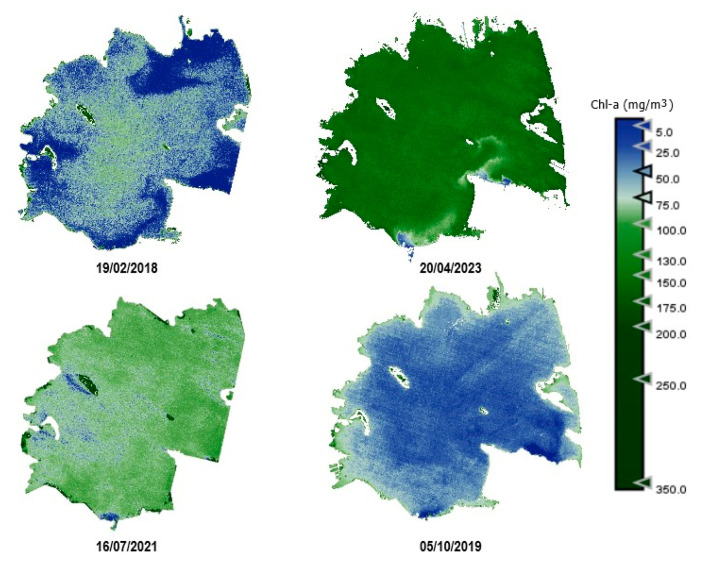
Estimation maps of Chl-*a* (μg/L). From left to right and up to down: winter, spring, summer, and autumn.

**Figure 11:**
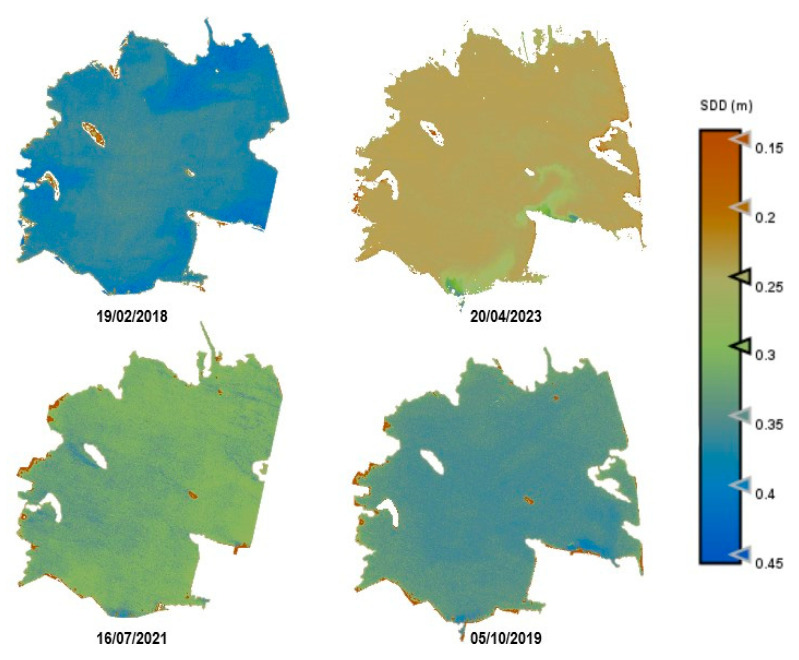
Estimation maps of SDD (m). From left to right and up to down: winter, spring, summer, and autumn.

**Figure 12:**
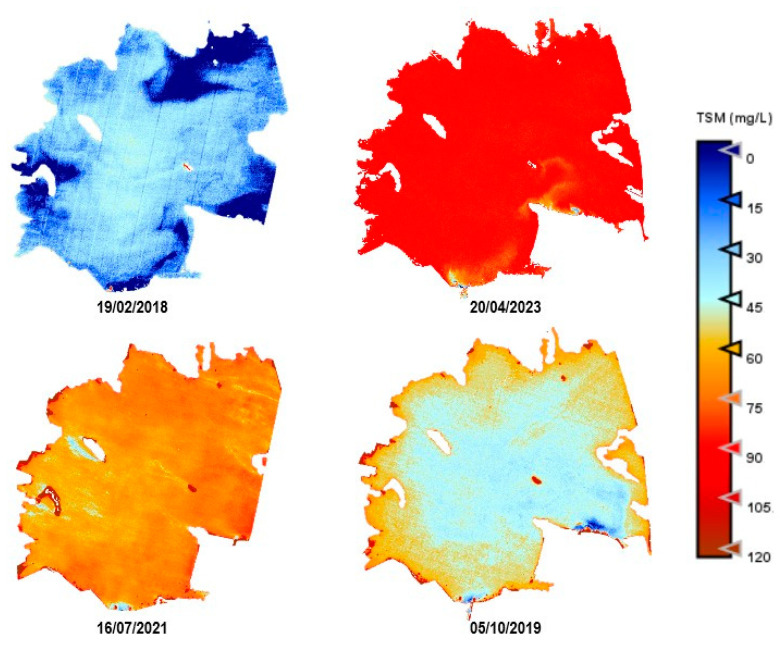
Estimation maps of TSM (mg/L). From left to right and up to down: winter, spring, summer, and autumn.

**Figure 13:**
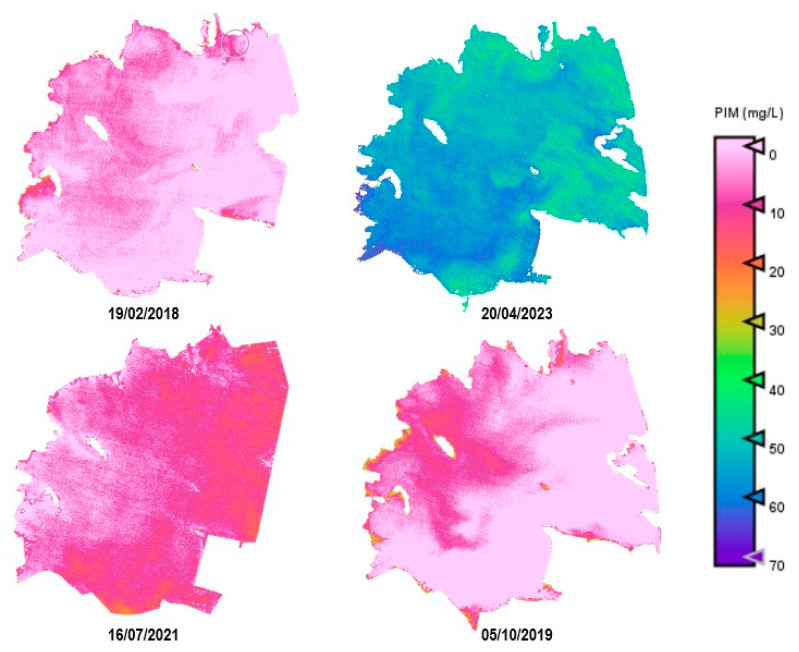
Estimation maps of PIM (mg/L). From left to right and up to down: winter, spring, summer, and autumn.

**Figure 14:**
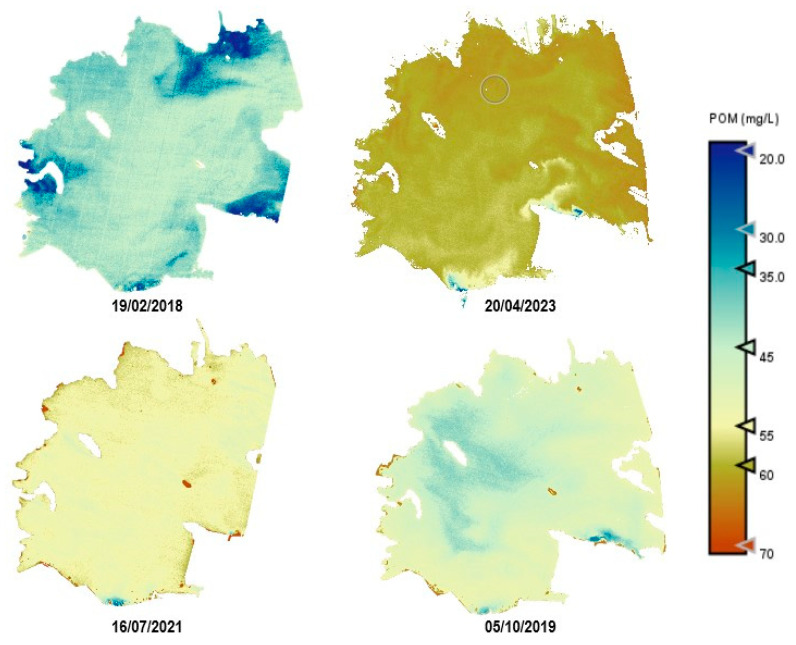
Estimation maps of POM (mg/L). From left to right and up to down: winter, spring, summer, and autumn.

As an example, Figure 10, Figure 11, Figure 12, Figure 13 and Figure 14 show thematic maps obtained for each parameter included in this study by applying the newly developed algorithms to PerúSAT-1 images of the *Albufera de València* (Table 10). Through the available PerúSAT-1 images, the selected dates are ordered based on the different seasons of the year (winter: 19 February 2018, spring: 20 April 2023; summer: 16 July 2021; autumn: 5 October 2019). It should be note that the hydrodynamic cycle could differ in other years, as it may be affected by weather, increases in flow, etc.

An important difference can be observed through the seasons. In April (spring season), we find higher values for Chl-*a*, TSM, and organic-inorganic fractions. As observed in the cross-correlation between all the variables in the first study, we can observe an existing inverse relationship between the highest values of these variables and the lowest values of SDD. It can also be observed that POM is predominant in the lagoon, but both fractions have the same trend over the seasons.

### 3.4. Spatial Resolution Capacity

**Figure 15:**
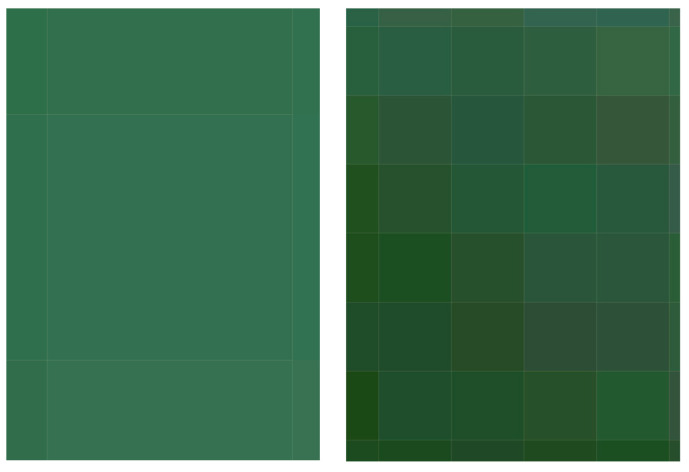
10 m pixel (**left**) of S2 image vs. 2.8 m pixel (**right**) of PerúSAT-1 image.

**Figure 16:**
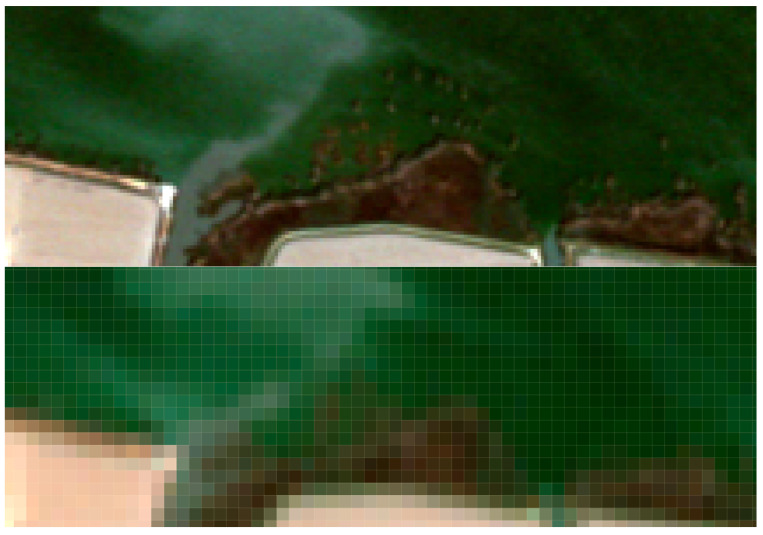
Subset of “Obera ditch” area for PerúSAT-1 product (**top**) and S2 product (**bottom**).

Figure 15 shows the performance of the spatial resolution in PerúSAT-1 (right) and S2 (left) pixels, and Figure 16 highlights the level of detail offered by this spatial resolution of 2.8 m (top) versus the 10 m one (bottom) in an irrigation area (*Obera* ditch) corresponding to the “south” point of our study. Border effect is less pronounced in the products offered by the PerúSAT-1 method, as the pixels are smaller, and it is possible to see more detail. Appendix A contains thematic maps obtained for each parameter included in this study, through the application of the algorithms summarized in Table 3 on S2 images and the algorithms summarized in Table 10 on PerúSAT-1 images. In addition, the thematic maps of Chl-*a* applied to the subset of Figure 16 are included.

Table 11 shows the performance comparison between the two satellites and the in situ data, using the algorithms summarized in Table 3 on S2 images and the algorithms summarized in Table 10 on PerúSAT-1 images. The comparison was made with the data from the last field campaign (20 April 2023), since both satellites passed over on that day. The data estimated with the PerúSAT-1 products correlate well with the field data.

## 4. Discussion

Authors such as [52,53] or [54], using SPOT, Landsat, and Modis satellites, determined that remote sensing data could be used as an effective predictor of the trophic status of inland water bodies. In our study, algorithms were developed for five water quality variables using the multispectral bands of the Peruvian satellite PerúSAT-1. For three variables, Chl-*a*, SDD, and PIM, the band combination was normalized difference (ND), and for TSM and POM, the band combination was simple ratio (SR). ND reduces the uncertainties in the estimation by excluding the seasonal solar azimuth differences and atmospheric contributions [55], and the implementation of spectral band ratios in the retrieval algorithms reduces irradiance, atmospheric, and air-water surface influences in the remotely sensed signal [56,57]. In the Albufera lagoon, the trophic state is always hypertrophic, with a large amount of suspended matter. Taking into consideration the low spectral resolution of the bands and the correlation between the variables studied, several algorithms share the same bands in their formulation. This is the case for Chl-*a*, SDD, and TSM, the most correlated variables, whose algorithms are based on the blue (B1) and infrared (B4) bands.

Chlorophyll-a concentrations vary between 21 and 331 µg/L, and the best algorithm was developed using the ND with infrared (B4) and blue (B1) (NRMSE = 16%). Similar studies demonstrated that by using the combination of the red and infrared bands in the Landsat TM and SPOT satellites, it is possible to determine the Chl-*a* in water bodies. For example, [58] used the TM3/TM1 ratio (R^2^ = 0.67) to estimate Chl-*a* in Pensacola Bay with a standard error of estimate of 1.55 µg/L. Similarly, [59] used in their study the ratio between blue and red for Landsat 5-TM (R^2^ = 0.72) in small lakes in Italy, with an RMSE of 1.3 mg/m^3^. Ref. [15] found that the ratio TM2/TM4 (R^2^ = 0.66) for Landsat images, also valid for SPOT images, was useful for estimating Chl-*a* using data from the Albufera lagoon.

Gitelson et al. [60,61] explain the principle behind these ratios as both bands corresponding to Chl-*a* absorption; Chl-*a* is directly proportional to the magnitude of the reflectance in the red band and inversely proportional to that in the blue or green band. When using the NIR band, results may be consistent, as the reflectance in this band may sometimes be less variable due to strong absorption by water [62,63].

With respect to SDD values, they range from 0.16 m to 0.42 m. As a result, the ND between infrared (B4) and blue (B1) (NRMSE = 15%) is the one that best fits the available data for quantifying this parameter. A study carried out by [64] in Argentina determined that using a combination of the blue band and the blue/NIR, green/NIR ratios for the OLI sensor (R^2^ = 0.84) provides a strong predictive relationship with SDD, with an RMSE of 0.56 m. While other authors, such as [65] with a standard error of estimation (SEE) of 0.28 m (R^2^ = 0.73), ref. [66] with an SEE of 0.292 m (R^2^ = 0.78), and [67] with an RMSE of 1.20 m (R^2^ = 0.72), concluded that SDD is strongly correlated with the responses in the blue and red bands of Landsat and MODIS data through the blue/red ratio in different lakes. As SDD decreases, the brightness in the red band tends to increase, which is explained by the positive correlation between red reflectance and particulate matter-inducing scattering. Dividing by the blue band, dominated by the absorbing effects of phytoplankton, normalizes the brightness in the red band. Furthermore, using the NIR band, rough atmospheric effects could be corrected [9].

Finally, TSM in situ presents a variation between 39 and 136 mg/L, with an organic fraction between 7 and 60 mg/L and an inorganic fraction between 2 and 75 mg/L. For these values, the best results are presented between the band ratios blue (B1) and infrared (B4) for TSM (NRMSE = 11%), red (B3) and infrared (B4) for POM (NRMSE = 9%), and the ND of red (B3) and blue (B1) for PIM (NRMSE = 15%). This may be due to the greater amount of POM in the study area, compared to PIM.

Most of the studies using SPOT and Landsat TM satellite data (e.g., ref. [68] with an RMSE of 25.77 mg/L (R^2^ = 0.94) for SPOT data and 25.31 mg/L (R^2^ = 0.98) for Landsat data) conclude from their results that the combination of green and red bands or blue and red bands presents high correlation coefficient values for the quantification of TSM. On the other hand, [69] demonstrated that through the ratio between blue and red with Landsat 5-TM, it is possible to determine TSM with an RMSE of 0.295 mg/L (R^2^ = 0.67). Likewise, ref. [13] achieved this detection using the combination of bands 3 (infrared) and 1 (green) of the SPOT satellite with an R^2^ of 0.93. Therefore, according to [70] and more recently [14], the wavelength between 700 and 800 nm is suitable for estimating TSM. The reason for the correlation, usually with red and NIR bands, is explained in [71] by the contribution of particulate matter, especially the inorganic component, to scattering in these bands. The algorithms for PIM detection used in [72] (R^2^ = 0.99) and [73] (R^2^ = 0.96) are very similar to those for TSM due to the above explanation. Far fewer studies have investigated PIM and POM fractions compared to TSM, making it difficult to find empirical algorithms that effectively separate these signals.

In our study, band 2 was not useful for the determination of any parameter. The study area has very eutrophic waters, and waters with high Chl-*a* (above 3–5 mg/m^3^) produce distinguishable spectral characteristics in the red and NIR regions of the reflectance spectrum [74]. Ref. [75] explains that inorganic mineral particles, unlike organic particles, have a greater refractive index and scatter light more in the NIR, which is better estimated with algorithms that include visible bands. However, ref. [20] explains that POM in the Albufera lagoon is better determined with bands close to the NIR, due to the high load of particulate matter present in the lagoon, as this high concentration produces a saturation in the visible bands, in favor of the red-edge and NIR bands.

**Figure A6:**
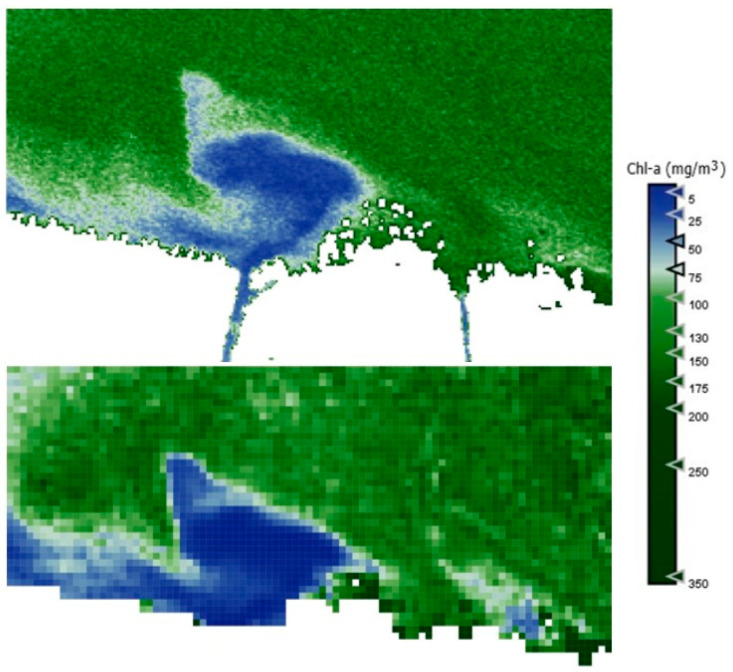
Subset of “Obera ditch” area for PerúSAT-1 Chl-*a* product (**top**) and S2 Chl-*a* product (**bottom**).

Regarding the comparison of spatial resolution, the fact that the PerúSAT-1 images appear more homogeneous may be due to the width of their bands. This width, as well as the fact that several algorithms use the same bands, can reduce their sensitivity to detecting small variations in the variables studied. However, the very high spatial resolution can be very useful when avoiding the border effect or the inclusion in a water mask of coastal areas with dense aquatic vegetation (see Appendix A, Figure A6). Additionally, when correlating the data obtained through the algorithms developed for the PerúSAT-1 products, the error improves slightly with respect to the data obtained with the S2 products. Nevertheless, S2 offers a better revisit time and has a better band configuration, only having lower spatial resolution than PerúSAT-1. Considering that PerúSAT-1 can change its observation geometry, which increases the revisit time, it would be interesting to combine the products obtained from both satellites to perform studies in specific locations, thus obtaining better results and more robust models. Validation of the atmospheric correction with the in situ Rrs data demonstrates that the method developed in this study can be useful for application to PeruSAT-1 imagery. The hydrodynamic cycle that occurs in the Albufera was once modified by human hands, and thematic maps (Figure 9, Figure 10, Figure 11, Figure 12 and Figure 13) show different phases of this cycle. Thus, there is a visible increase in Chl-*a*, TSM, and its organic/inorganic fractions during spring. This increase coincides with the time when the fields are flooded for rice planting, possibly due to runoff from the fertilizers used in the crops. It can also be observed that the parameters begin to decrease in summer when a nutrient depletion occurs after the spring peak of primary production. Regarding the spatial distribution, it is observed that in the water inlet areas, where Chl-*a* concentration is lower, there are higher SDD values. In these same entrance areas, inorganic solids predominate with respect to the center of the lagoon, where, since there is a higher Chl-*a* concentration, organic solids predominate, and, in addition, there are lower SDD values.

## 5. Conclusions

To sum up, our study has identified that through the ND with infrared (B4) and blue (B1) bands of PerúSat-1, it is possible to determine the amount of Chl-*a* in eutrophic lakes, with a relative error of 16% (NRMSE), and to calculate the SDD with a relative error of 15% (NRMSE). Regarding TSM and its inorganic plus organic fraction, our results have demonstrated that we could use band ratios blue (B1) and infrared (B4) for TSM, red (B3) and infrared (B4) for POM, and the ND of red (B3) and blue (B1) for PIM, with relative errors of 11%, 15%, and 9% (NRMSE), respectively.

Due to an existing correlation between Chl-*a* and SDD in the eutrophic waters of the Albufera lagoon, the algorithms to determine both variables through PerúSat-1 images are very similar, as they use the same combination of bands.

Finally, the development of those new algorithms obtained through the combination of in situ data and data extracted from the PerúSat-1 satellite leads to the possibility of having more detailed information to study the spatial dynamics that occur in the Albufera lagoon, especially with the greatest spatial resolution that it offers. PerúSat-1 is a tool with the potential to be used in water quality studies, especially in hypertrophic waters.

## Figures and Tables

**Table 1:** Summary of the field campaigns and image acquisition data. N is the number of samples per campaign.

Year	2018	2018	2019	2021	2023
**Field Campaign**	16 February 2018	23 May 2018	8 October 2019	14 July 2021	20 April 2023
**Image Acquisition**	19 February 2018	22 May 2018	5 October 2019	16 July 2021	20 April 2023
**N**	5	5	4	4	4

**Table 2:** PerúSAT-1 spectral bands description.

Spectral Band	Range (nm)	Spatial Resolution (m)
B1 (blue)	450–520	2.8
B2 (green)	530–590	2.8
B3 (red)	630–700	2.8
B4 (NIR)	752–885	2.8
Panchromatic	450–750	0.7

**Table 3:** Summary of equations used to calculate each parameter with Sentinel-2 images. TBDO: Triband model [48].

Parameter	Formula	R^2^	RMSE	Author
*Chl-a*	104.1 x TBDO^2^ + 221.1 x TBDO + 2 *TBDO = R_740_ x ((1/R_665_) − (1/R_705_))*	0.99	40 µg/L	[27]
*SDD*	0.224 x (R_560_/R_704_) + 0.0836	0.67	0.07 m	[49]
*TSM*	705.98 x R_783_ x R_705_/R_490_	0.91	26.64 mg/L	[20]
*PIM*	259.4 x R_705_	0.79	25.83 mg/L	[20]
*POM*	40.48 x R_783_/R_490_	0.91	14.42 mg/L	[20]

**Table 4:** Correlation coefficients and data points (in parenthesis).

*p* < 0.001	SDD	TSM	PIM	POM
Chl-*a*	−0.845 (22)	0.174 (14)	−0.191 (14)	0.305 (14)
SDD		−0.321 (14)	−0.156 (14)	−0.292 (14)
TSM			0.679 (14)	0.873 (14)
PIM				0.455 (14)

**Table 5:** Best algorithms for Chl-*a* estimation.

Band Comb. (x)	Formula (y = Chl-*a* in µg/L)	R²	RMSE (µg/L)	NRMSE (%)	MAE	Bias
$\frac{\left(B4-B1\right)}{\left(B4+B1\right)}$	y = 323.77x + 198.95	0.76	50.8	16	43.9	−0.0050
$\frac{\left(B4-B2\right)}{\left(B4+B2\right)}$	y = 468.33x + 345.39	0.72	54.6	18	44.6	0.0038
$\frac{B4}{\;B1}$	y = 169.2x + 3.3682	0.70	56.9	18	46.1	0.0007
$\frac{B4}{B2}$	y = 420.28x − 35.035	0.70	57.7	19	45.7	−0.0018
$\frac{\left(B4-B3\right)}{\left(B4+B3\right)}$	y = 303.82x + 144.99	0.67	59.5	19	44.9	−0.0013

**Table 6:** Best algorithms for SDD estimation.

Band Comb. (x)	Formula (y = SDD in m)	R²	RMSE (m)	NRMSE (%)	MAE	Bias
$\frac{\left(B4-B1\right)}{\left(B4+B1\right)}$	y = −0.2539x + 0.2608	0.76	0.039	15	0.031	0.00003
$\frac{\left(B4-B2\right)}{\left(B4+B2\right)}$	y = −0.37x + 0.1448	0.73	0.041	16	0.035	−0.00002
$\frac{B4}{B2}$	y = 0.0549x + 0.1449	0.71	0.044	17	0.036	−0.00015
$\frac{B4}{B1}$	y = −0.1315x + 0.4124	0.69	0.045	17	0.038	−0.00006
$\frac{\left(B4-B3\right)}{\left(B4+B3\right)}$	y = −0.212x + 0.303	0.53	0.055	21	0.040	−0.00001

**Table 7:** Best algorithms for TSM estimation.

Band Comb. (x)	Formula (y = TSM in mg/L)	R²	RMSE (mg/L)	NRMSE (%)	MAE	Bias
$\frac{B1}{B4}$	y = −38.259x + 129.38	0.85	11.1	11	7.0	0.0033
$\frac{\left(B4-B1\right)}{\left(B4+B1\right)}$	y = 97.698x + 84.465	0.84	11.4	12	6.7	0.0001
$\frac{\left(B4-B2\right)}{\left(B4+B2\right)}$	y = 130.16x + 124.12	0.83	11.5	12	6.7	−0.004
$\frac{B2}{B4}$	y = −22.907x + 133.88	0.81	12.4	13	8.9	−0.0007
$\frac{B4}{B1}$	y = 42.293x + 33.256	0.72	15.0	15	10.6	−0.0001

**Table 8:** Best algorithms for PIM estimation.

Band Comb. (x)	Formula (y = PIM in mg/L)	R²	RMSE (mg/L)	NRMSE (%)	MAE	Bias
$\frac{\left(B3-B1\right)}{\left(B3+B1\right)}$	y = 116.08x + 31.36	0.76	10.7	15	8.0	0.0002
$\frac{B3}{\;B1}$	y = 58.06X − 30.14	0.76	10.9	15	8.0	−0.0006
$\frac{B1}{B3}$	y = −52.368x + 86.82	0.75	11.0	15	8.3	−0.0005
$\frac{B4}{B2}$	y = 80.777x − 12.475	0.71	11.9	16	8.8	−0.0002
$\frac{\left(B4-B2\right)}{\left(B4+B2\right)}$	y = 92.683x + 61.954	0.70	12.0	16	8.9	0.0001

**Table 9:** Best algorithms to estimate POM.

Band Comb. (x)	Formula (y = POM in mg/L)	R²	RMSE (mg/L)	NRMSE (%)	MAE	Bias
$\frac{B3}{B4}$	y = −18.775x + 70.925	0.87	4.7	9	4.2	−0.0003
$\frac{\left(B4-B3\right)}{\left(B4+B3\right)}$	y = 62.287x + 51.562	0.73	6.8	13	5.7	−0.0002
$\frac{B4}{B3}$	y = 33.551x + 16.397	0.56	8.7	16	7.0	0.0004
$\frac{B1}{B4}$	y = −13.507x + 67.541	0.49	9.4	18	7.0	−0.0006
$\frac{\;B2}{B4}$	y = −7.6115x + 67.808	0.41	10.1	19	7.2	0.0002

**Table 10:** Summary of best algorithms obtained.

Variable	Range	n	Equation	R²	RMSE	NRMSE	MAE	Bias
Chl-*a* (µg/L)	21–331	21	Chl-*a* (µg/L) = 323.77 ($\frac{\left(B4-B1\right)}{\left(B4+B1\right)}$) + 198.95	0.76	50.8	16	43.9	−0.0049
SDD (m)	0.16–0.42	22	$\mathrm{SDD}\;(m)=-0.2539\;(\frac{\left(B4-B1\right)}{\left(B4+B1\right)}$) + 0.2608	0.76	0.039	15	0.031	0.00003
TSM (mg/L)	50–136	12	$\mathrm{TSM}\;(\mathrm{mg}/L)=-38.259\;(\frac{B1}{B4}$) + 129.38	0.85	11.1	11	7.0	0.0033
PIM (mg/L)	2–75	12	PIM (mg/L) = 116.08 ($\frac{\left(B3-B1\right)}{\left(B3+B1\right)}$) + 31.36	0.76	10.7	15	8.0	0.0002
POM (mg/L)	7–60	12	$\mathrm{POM}\;(\mathrm{mg}/L)=-18.775\;(\frac{B3}{B4}$) + 70.925	0.87	4.7	9	4.2	−0.0003

**Table 11:** Statistical results of each sensor and variable validation.

Parameter	Satellite	R^2^	RMSE	NRMSE	MAE	Bias
*Chl-a*	PerúSAT-1	0.88	46.5	16	42.0	24.6
	S2	0.84	63.0	21	43.6	17.5
*SDD*	PerúSAT-1	0.86	0.036	15	0.031	−0.006
	S2	0.90	0.053	22	0.040	0.039
*TSM*	PerúSAT-1	0.78	19.1	20	15.8	−7.3
	S2	0.70	33.6	64	27.4	−27.4
*PIM*	PerúSAT-1	0.02	17.2	38	14.9	0.7
	S2	0.56	34.2	76	30.7	−30.7
*POM*	PerúSAT-1	0.95	5.4	10	5.1	−1.5
	S2	1.00	11.4	22	10.4	7.4

**Figure A1:**
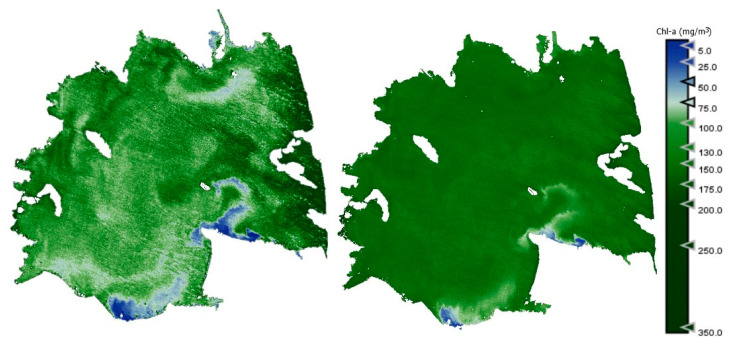
Estimation maps of Chl-*a*. Acquisition data: 20 April 2023. Left: S2 image; right: PerúSAT-1 image.

**Figure A2:**
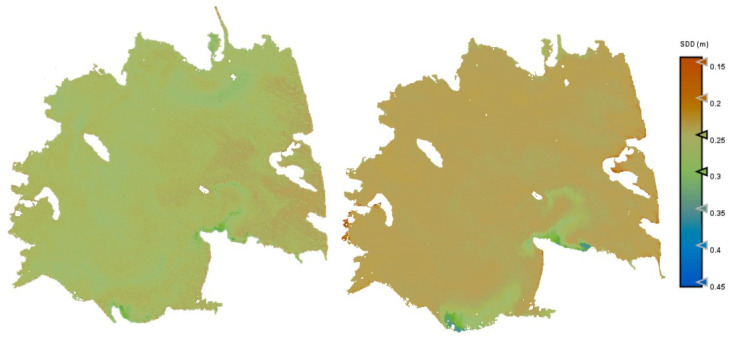
Estimation maps of SDD. Acquisition data: 20 April 2023. Left: S2 image; right: PerúSAT-1 image.

**Figure A3:**
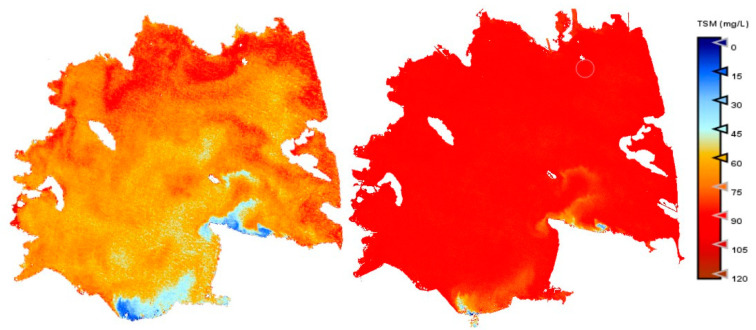
Estimation maps of TSM. Acquisition data: 20 April 2023. Left: S2 image; right: PerúSAT-1 image.

**Figure A4:**
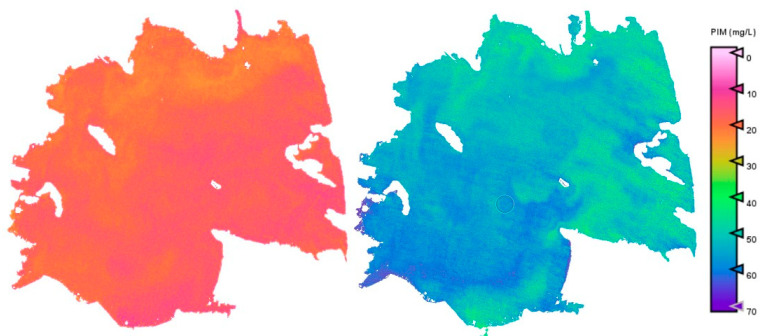
Estimation maps of PIM. Acquisition data: 20 April 2023. Left: S2 image; right: PerúSAT-1 image.

**Figure A5:**
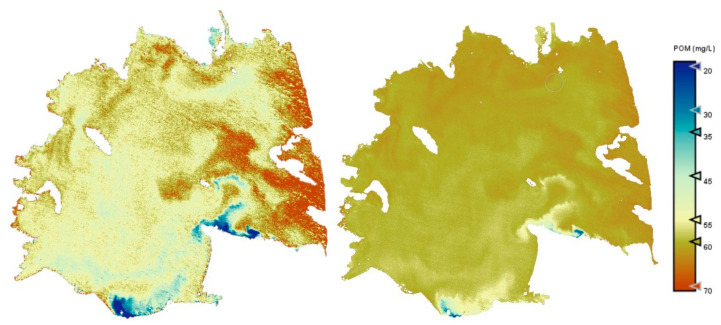
Estimation maps of POM. Acquisition data: 20 April 2023. Left: S2 image; right: PerúSAT-1 image.

## Data Availability

Data are contained within the article.

## Abbreviations

The following abbreviations are used in this manuscript:

Chl-*a*	Chlorophyll *a*
SDD	Secchi disk depth
TSM	Total Suspended Matter
PIM	Particulate inorganic matter
POM	Particulate organic matter
S2	Sentinel-2
TOA	Top of atmosphere
BOA	Bottom of atmosphere
ND	Normal difference
SR	Simple ratio

## Appendix A

Figures showing the thematic maps obtained for each parameter included in this study, through the application of algorithms summarized in Table 3 on S2 images and the algorithms summarized in Table 10 on PerúSAT-1 images.

The improvement in heterogeneity offered by PerúSAT-1 is evident in Figure A6, allowing us to see the gradual changes and therefore the spatial distribution at different points of the lagoon.
